# Identification of A Novel Compound Heterozygous Mutation in *BBS12* in An Iranian Family with Bardet-Biedl Syndrome Using Targeted Next Generation Sequencing

**DOI:** 10.22074/cellj.2018.5012.

**Published:** 2018-03-18

**Authors:** Emad Nikkhah, Reza Safaralizadeh, Javad Mohammadiasl, Maryam Tahmasebi Birgani, Mohammad Ali Hosseinpour Feizi, Neda Golchin

**Affiliations:** 1 Department of Animal Biology, Faculty of Natural Science, University of Tabriz, Tabriz, Iran; 2 Department of Medical Genetics, School of Medicine, Ahvaz Jundishapur University of Medical Sciences, Ahvaz, Iran; 3 Noor Genetics Lab, Ahvaz, Iran

**Keywords:** Bardet-Biedl Syndrome, BBS12, Mutation, Sequence Analysis

## Abstract

Bardet-Biedl syndrome (BBS) is a pleiotropic and multisystemic disorder characterized by rod-cone dystrophy, polydactyly, learning difficulties, renal abnormalities, obesity and hypogonadism. This disorder is genetically heterogeneous. Until now, a total of nineteen genes have been identified for BBS whose mutations explain more than 80% of diagnosed cases. Recently, the development of next generation sequencing (NGS) technology has accelerated mutation screening of target genes, resulting in lower cost and less time consumption. Here, we screened the most common BBS genes (*BBS1-BBS13*) using NGS in an Iranian family of a proposita displaying symptoms of BBS. Among the 18 mutations identified in the proposita, one (*BBS12 c.56T>G and BBS12 c.1156C>T*) was novel. This compound heterozygosity was confirmed by Sanger sequencing in the proposita and her parents. Although our data were presented as a case report, however, we suggest a new probable genetic mechanism other than the conventional autosomal recessive inheritance of BBS. Additionally, given that in some Iranian provinces, like Khuzestan, consanguineous marriages are common, designing mutational panels for genetic diseases is strongly recommended, especially for those with an autosomal recessive inheritance pattern.

## Introduction

Bardet-Biedl syndrome (BBS, MIM#209900) is a rare genetic condition diagnosed with a wide range of major and minor symptoms including learning difficulties, obesity, rod-cone dystrophy, polydactyly, genital anomalies and renal abnormalities. In addition, other symptoms including speech and developmental delay, diabetes, dental anomalies, congenital heart disease, brachydactyly/syndactyly, ataxia, deafness and ansomia have also been reported (1).

Usually, BBS can be diagnosed by the presence of at least four major features or the combination of three major and at least two minor features (2). The incidence of BBS varies among different populations and is increased in regions with a high level of consanguinity. For instance, in North America and Europe, the prevalence of BBS is estimated around 1/160,000 (3) while this frequency rises to 1/13,500 in Kuwait, most likely due to the high level of consanguinity and founder effects (4-6).

The syndrome shows an autosomal recessive inheritance pattern, however, oligogenic patterns have also been observed (7, 8). Until now, a total of nineteen gene shave been identified for BBS which play specific roles in cilium biogenesis and function (8-12). These genes are *BBS1, BBS2, BBS3 (ARL6), BBS4, BBS5, BBS6 (MKKS), BBS7, BBS8 (TTC8), BBS9 (PTHB1), BBS10, BBS11(TRIM32), BBS12, BBS13 (MKS1), BBS14 (CEP290), BBS15 (C2orf86), BBS16 (SDCCAG8), BBS17 (LZTFL1), BBS18 (BBIP1) and BBS19 (IFT27) *(8, 12). Mutations in this gene panel explain more than 80% of identified cases (7, 13-15). Furthermore, the distribution of BBS-causative mutations varies among different geographical regions; *BBS1* and *BBS10* are the most frequently mutated genes in European and North American populations, whereas BBS2, BBS4, BBS5 and BBS12 are common in Middle East and North Africa (7, 16-18).

Recently, robust genomic analysis including homozygosity mapping and high-throughput sequencing holds the promise of identifying novel causative mutations in such a heterogeneous condition (1). Targeted next generation sequencing (NGS) is one of the favorite strategies for medical geneticists to screen known genes across the whole genome affordably (19). The present study was aimed to screen *BBS* genes in an Iranian female with symptoms of BBS. Targeted NGS identified a novel compound heterozygous mutation in *BBS12*.

## Case report

**Fig.1:**
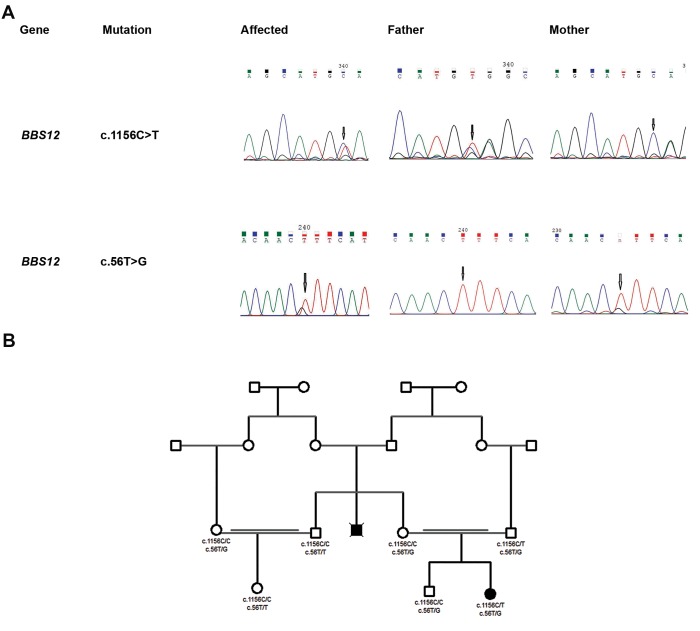
The patient had undergone surgery for correcting the postaxial polydactyly at the age of one. The above photograph was taken with the consent of the parents of patient at the Noor Genetics Laboratory.

A 13-year-old Iranian female was admitted to the Noor Medical Genetic Clinic for truncal obesity and blindness. She was the first offspring of a consanguineous marriage. Her parents were healthy as was her younger brother. Initial evaluation confirmed polydactyly (specifically hexadactyly) of all four limbs, congenital heart disease, blindness and obesity. We also found hypothyroidism and dental anomalies such as crowding of the teeth, however urinalysis, complete blood count and renal function tests were found to be normal. She had a rather normal facies and hearing impairment was not identified. She had experienced normal maturation at puberty and showed secondary sexual characteristics such as pubic hair and regular menses. At one year of age, she had undergone surgery for correcting the postaxial polydactyly of the four limbs (Fig .1). 

She had learned to walk and speak at the age of two but had difficulty in finding words. Learning disabilities was noted at the age of eight, when she had also started to complain of night blindness. Two years later, at the age of ten, she had become blind. There was a family history of death due to renal dysfunction in her maternal uncle, who had displayed similar phenotypic characteristics. According to the clinical background and consanguineous nature of the relationship of her parents, BBS was diagnosed by the physician and therefore genetic screening was undertaken. 

### Patient recruitment 

This study was Ethically approved by Tabriz University, Tabriz, Iran. All the participants signed an informed consent prior to joining the project. We studied all the available members who were informative for tracking the origin of mutation(s) in the pedigree, namely the proposita, father, mother, brother and the uncle’s nuclear family (i.e. uncle’s wife and daughter). 

### DNA extraction

Blood sample (5 ml) was collected in ethylenediaminetetraacetic acid (EDTA)-containing tubes from each participant and genomic DNA was extracted from peripheral blood samples using the salting out method (20). The quality of extracted DNA was checked by 1% agarose gel (KBC, Iran) electrophoresis followed by ethidium bromide staining (Merck, Germany). The optical density of extracted DNA was also examined at 260 nm and 280 nm using the Nanodrop Analyzer (ND1000) spectrophotometer (Thermo Fisher Scientific, USA) to evaluate the purity of each sample and detect possible contamination.

### Targeted next generation sequencing

DNA extracted from the proposita was submitted to BGI (BGI-clinical laboratories, China) for whole genome amplification using a custom designed chip to capture the genes *BBS1-BBS13* to identify potentially pathogenic variants in these genes.

### In silico mutation analysis 

Criteria used to assign a mutation as novel and pathogenic were previously described by Chen et al. (21). Accordingly, the genomic variants were considered as novel if not previously reported in dbSNP or the literature. Polyphen (http://genetics.bwh.harvard.edu/pph2/), PROVEAN (<uri>http:// provean.jcvi.org/index.php</uri>) and SIFT (<uri>http://sift.bii.a-star. edu.sg/</uri>) were used to predict if any variant is pathogenic by potentially affecting the protein structure. 

Additionally, to evaluate if the novel mutation had occurred in a conserved domain of a target gene, the protein sequence of that gene were obtained for different species from the NCBI protein database (<uri>http://www.ncbi.nlm.nih.gov/ protein/</uri>) and aligned using ClustalW2 (<uri>http://www.ebi.ac.uk/ Tools/clustalw2</uri>). The novel variants were eventually traced in the family of the proposita to uncover their parental origin.

### Polymerase chain reaction and Sanger sequencing

To confirm the mutations detected based on targeted NGS, Sanger sequencing of the regions containing the mutations was undertaken. First, genomic DNA was amplified with polymerase chain reaction (PCR) using specific primers flanking the mutation regions. The primer sequences and their related amplicon are illustrated (Table 1). PCR reactions were carried out in a total volume of 25 µl containing 1X reaction buffer (Merck, Germany), 0.5 µg of genomic DNA template, 1.5 U of Taq DNA polymerase (KBC, Iran), 2 pmol/L of each primer (Macrogen, Korea) and 0.25 mM of each dNTP (KBC, Iran). PCR cycling conditions were 5 minutes denaturation at 95°C for initial denaturation, 35 cycles of denaturation at 95°C for 30 seconds, annealing at 60°C for 30 seconds and extension at 72°C for 30 seconds, followed by a final extension at 72°C for 2 minutes. Additionally, a negative control (no template DNA sample) was included in all PCR reactions. PCR products were then analyzed on a 1.5% agarose gel dyed with ethidium bromide (2%) and product bands were visualized under ultraviolet light (UV Tec, USA). Finally, using the same primers, Sanger sequencing was undertaken by the means of Big Dye Terminators (Applied Bio systems 3130 Genetic Analyzer, Applied Bio systems, Foster City, CA, USA). 

### A novel pathogenic variant in *BBS12* were detected in targeted NGS of the proposita 

Targeted NGS was conducted on 13 common *BBS* genesof the proposita. A total of twenty two genetic variants weredetected, of which one was novel (Table 2). The novel variant *BBS12* c.56T>G (p.Leu19Arg) and *BBS12* c.1156C>T (p.Arg386Trp) occurred in exon 2 of *BBS12* and the propositawas heterozygote for both variants. The frequency of thesetwo variants in single nucleotide polymorphism database(dbSNP), HapMap, 1000 Genomes and BGI’s database is very low (<1%) (Table 2). 

**Fig.2:**
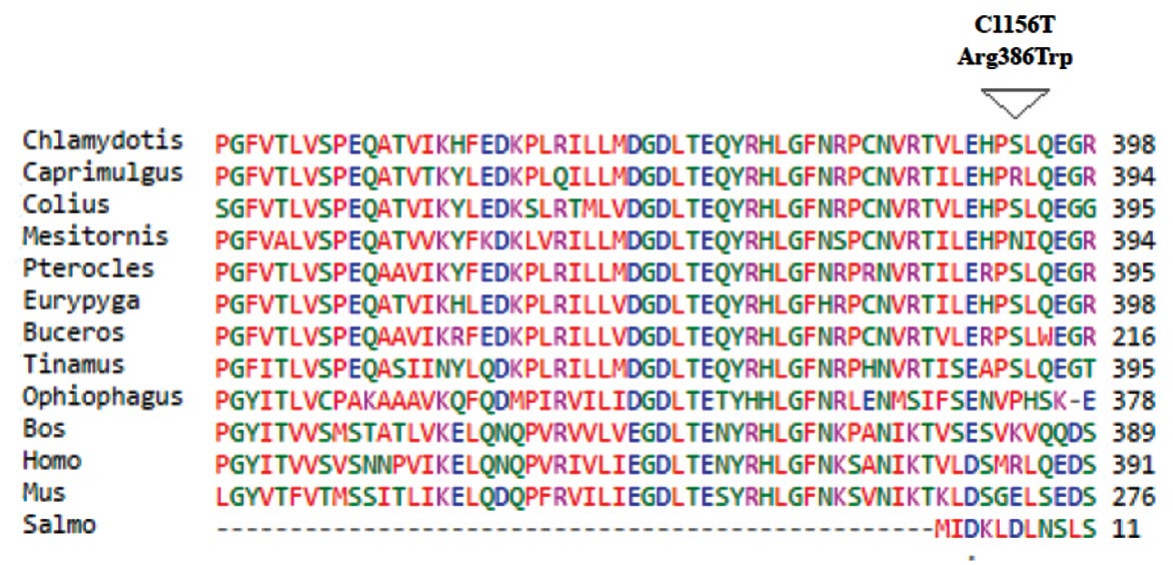
Sequence alignment of BBS12 of several species showing the conserved position of Leu19 and the non-conserved Arg386.

In silico mutation analysis using SIFT, PolyPhen and PROVEAN predicted that the mutation *BBS12* c.56T>G (p.Leu19Arg) is damaging and localized in a conserveddomain of BBS12. However the mutation *BBS12* c.1156C>T (p.Arg386Trp) is predicted to be either damaging or benignand also not confined in a conserved domain of *BBS12* (Fig .2). No damaging mutations were found in other *BBS* Genes. In specific, defects in *BBS12* cause BBS type 12. Thereis ample evidence showing the causal relationship of *BBS12* variants with BBS, however, in the Iranian population, only two studies have reported this relationship (Table 3). 

**Table 1:** List of the primer sets and related amplicons


Mutation	Primer	Sequence (5ˊ-3ˊ)	PCR product (bp)

BBS12 c.56 T>G	bbs12ex1-1F584	CCTCTGTTGGGTGGAGTGTT	584
	bbs12ex1-1R584	ACAAAAGTTTAAGCCTTCTGACA	
BBS12 c. 1156 C>T	bbs12ex1-3F500	TGAGTCATGGAGATCACAGCA	500
	bbs12ex1-3R500	CACACTGCCATTCACTGAGC	


PCR; Polymerase chain reaction.

**Table 2:** Variants identified in all targeted BBS genes in the proposita


Gene	Mutation name	SubRegion	Nucleotidechange	RS ID	Het/Hom	Mutation type	Freq_HapMap	Freq_dbSNP	Clinical significance

BBS4	c.77-6G>A	IN2	c.77-6G>A	rs8033604	Hom	Splice	1	0.908	Benign
	p.Phe302Phe	EX12/CDS12	c.906T>C	rs12914333	Hom	Synonymous	1	0.94	Benign
	p.Ile354Thr	EX13/CDS13	c.1061T>C	rs2277598	Hom	Missense	0.051	0.203	Likely benign
BBS6	p.Pro39Pro	EX3/CDS1	c.117C>T	rs16991547	Het	Synonymous	0.299	0.323	Likely benign
	p.Ile178Ile	EX3/CDS1	c.534C>T	rs17852625	Het	Synonymous	0	0.284	Other
	p.Arg517Cys	EX6/CDS4	c.1549C>T	rs1547	Het	Missense	0.307	0.287	Likely benign
	p.Gly532Val	EX6/CDS4	c.1595G>T	rs1545	Het	Missense	0.307	0.286	Likely benign
BBS10	p.Pro539Leu	EX2/CDS2	c.1616C>T	rs35676114	Het	Missense	0	0.068	Likely benign
BBS11	p.Val418Val	EX2/CDS1	c.1254G>A	rs1661300	Het	Synonymous	0.228	0.19	Other
BBS12	p.Leu19Arg	EX2/CDS1	c.56T>G	Novel	Het	Missense	0	0	-
	p.Arg386Trp	EX2/CDS1	c.1156C>T	rs202225266	Het	Missense	0	0	uncertain significance
	p.Arg386Gln	EX2/CDS1	c.1157G>A	rs309370	Hom	Missense	0.382	0.229	Benign
	p.Val460Val	EX2/CDS1	c.1380G>C	rs13135766	Het	Synonymous	0	0.198	Likely benign
	p.Gly466Gly	EX2/CDS1	c.1398C>T	rs2292493	Het	Synonymous	0.46	0.399	Benign
	p.Asp467Asn	EX2/CDS1	c.1399G>A	rs13135778	Het	Missense	0.007	0.194	Likely benign
	p.Cys470Cys	EX2/CDS1	c.1410C>T	rs13135445	Het	Synonymous	0	0.244	Likely benign
	p.Gln624Gln	EX2/CDS1	c.1872A>G	rs13102440	Het	Synonymous	0	0.193	Likely benign
INPP5E(JBTS1)	p.Pro324Pro	EX3/CDS3	c.972A>G	rs10870199	Het	Synonymous	0.277	0.21	Other
	p.Thr416Thr	EX5/CDS5	c.1248T>C	rs10781542	Het	Synonymous	0.321	0.471	Other
	p.Gly428Gly	EX6/CDS6	c.1284T>C	rs10870194	Het	Synonymous	0.313	0.47	Other
	p.His507His	EX7/CDS7	c.1521C>T	rs10870188	Het	Synonymous	0	0.215	Other
	p.Gly598Gly	EX9/CDS9	c.1794G>T	rs33982662	Het	Synonymous	0	0.3	Other


dbSNP; Single nucleotide polymorphism database.

**Table 3:** BBS12 variation identified in different populations


Nucleotide change	Amino acid change	Type of variation	Ethnic origin	References

c.56T>G	p.L19R	Missense	Iranian	This study
c.1156C>T	p.R386W	Missense	Iranian	This study
c.1156_1157 CG>TA	p.R386X	Nonsense	Iranian	(22)
c.1507G>A	p.V503M	Missense	Egyptian	(23)
c.1560G>A	p.W520X	Nonsense	Tunisian	(21)
c.1589T>C	p.L530P	Missense	Pakistani	(24)
c.1619G>T	p.G540D	Missense	Gypsy	(25)
c.1620 G>A	p.G540D	Missense	Caucasian	(26)
c.1993_1996del	p.V665Lfs*14	Deletion	Arabs	(27)
c.2019del	p.W673Cfs*7	Deletion	Iranian	(22)
c.2023C>T	p.R675X	Nonsense	Caucasian	(21)
c.2103C> A	p.S701X	Nonsense	Pakistani	(18)
c.3232C>T	p.P108L	Missense	Caucasian	(26)


**Fig.3:**
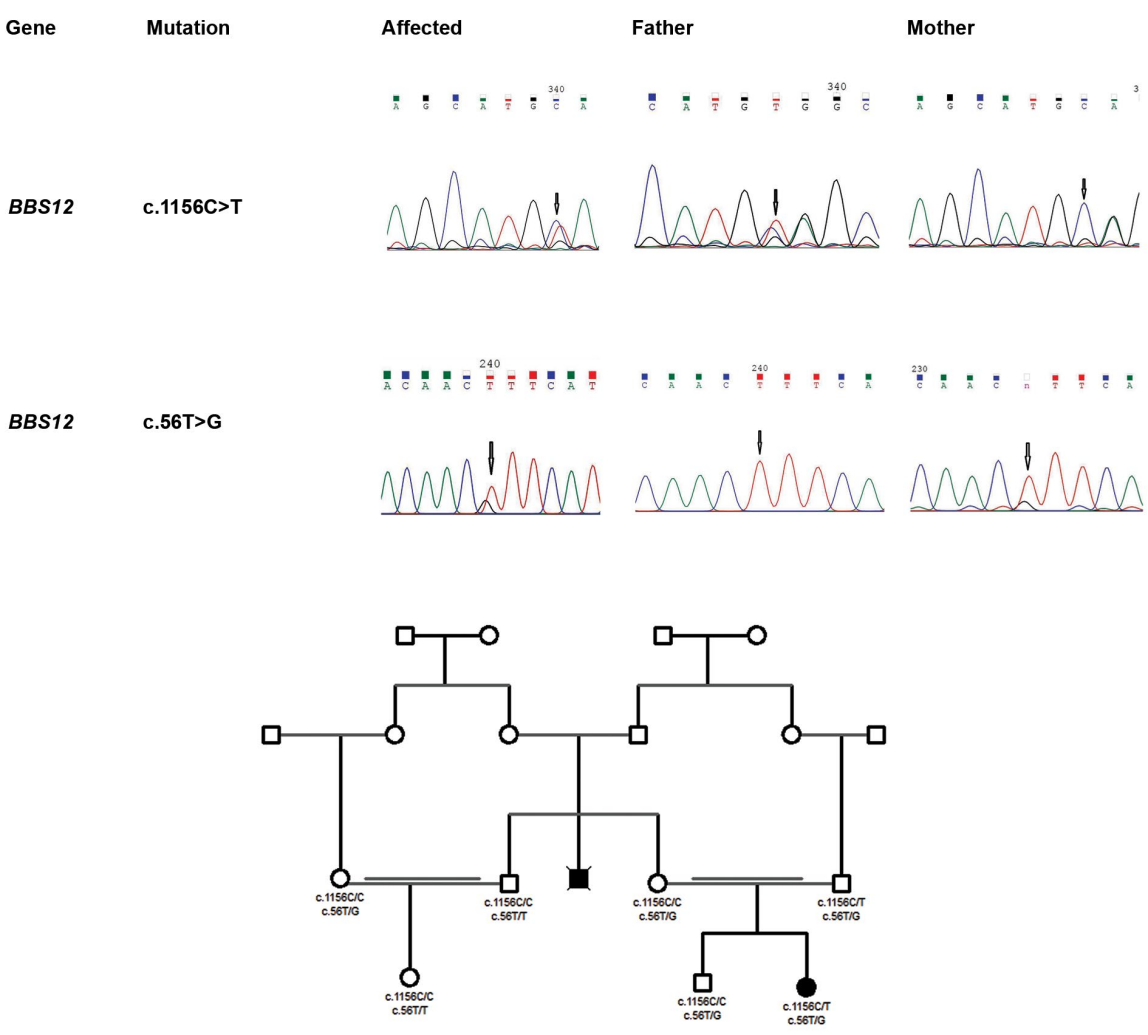
Sequence analysis and pedigree of the Bardet-Biedl syndrome case. A. Sequence analysis of c.1156C>T and c.56T>G in BBS12 of the proposita and her parents. The proposita carries both mutations as a compound heterozygote and B. Pedigree of the Bardet-Biedl syndrome case: proposita has received c.1156C>T from her father and c.56T>G from her mother.

### Sanger sequencing revealed that the proposita carries the novel variants as a compound heterozygote

Sanger sequencing was carried out on the proposita and her family to validate the NGS-based variants and their parental origin. We found that the affected girl was compound heterozygote for the two variants; the mother and the father harbored BBS12 c.56T>G and BBS12 c.1156C>T respectively. The variant status in the maternal uncle’s nuclear family members is shown (Fig .3A). The BBS12 c.56T/G variant originates from a maternal ancestor (Fig .3B).

## Discussion

This case report provided data of a genetic screening of BBS in an Iranian proposita suffering from this syndrome. Due to the heterogeneous nature of BBS, targeted NGS was applied to screen any causal mutations in thirteen BBS (1-13) genes. We identified a novel BBS12 mutations as compound heterozygote c.56T>G (p.Leu19Arg) and c.1156C>T (p.Arg386Trp), this mutation was not previously reported in SNP database. 

The *BBS12* gene, located on 4q27, is one of the key genes involved in pathogenicity of BBS. The gene structurally only contains two exons (25). The protein encoded by *BBS12* is not only part of a complex involved in cilia movement, but it is also involved in adipocyte differentiation. Three proteins BBS6, BBS10 and BBS12 are key members of the chaperonin complex. This complex contributes to cilia movement and therefore its defect reduces the mobility of the cilia and result in BBS symptoms including retinopathy, polydactyly, mental retardation and obesity (12). 

Using whole exome sequencing, the mutation profile of *BBS* genes in 14 Iranian families with Bardet-Biedl syndrome was reported by Fattahi et al. (22). They found five novel mutations of which most (28.6% of patients) occurred in *BBS2* with others occurring in *BBS4, BBS7* and *BBS12*. This finding was in contrast to that reported in European and North American populations where *BBS1* and *BBS10* were the most frequently mutated genes accounting for 23% and 20% of BBS patients respectively. It is important to mention that *BBS12* c.1156C>T sequence variant was also observed in the study by Fattahi et al. (22) but in a more complex form of *BBS12* c.1156_1157CG>TA, resulting in a nonsense mutation. In another study on 23 Iranian family members with BBS children, BBS was linked to markers at 3p13 p12where the *BBS3* gene is located (28).

## Conclusion

We should stress that previous studies on Iranian BBS patients including ours have limited sample sizes which may be due to the rare prevalence of the disease in population, however, all have been informative on the Iranian population. Additionally, given that some Iranian provinces like Khuzestan have a higher rate of consanguineous marriages, designing population- specific mutational panels for genetic diseases especially those with an autosomal recessive inheritance pattern are strongly recommended. Finally, allelic and locus heterogeneity of diseases such as BBS further emphasizes the benefits of NGS technology to genetically confirm the clinical diagnosis.
